# Iron-Containing Seed Particles Enhance α-Pinene Secondary Organic Aerosol Mass Concentration and Dimer Formation

**DOI:** 10.1021/acs.est.4c07626

**Published:** 2024-09-10

**Authors:** Natasha M. Garner, Jens Top, Fabian Mahrt, Imad El Haddad, Markus Ammann, David M. Bell

**Affiliations:** PSI Center for Energy and Environmental Sciences, Paul Scherrer Institute, 5232 Villigen, Switzerland

**Keywords:** terpene oxidation, α-pinene, dimers, HOMs, peroxides, particulate iron, Fenton chemistry, SOA formation, SOA aging, chamber studies

## Abstract

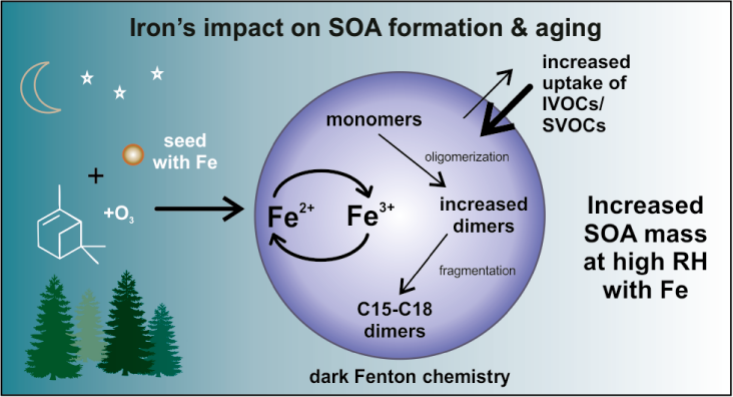

Secondary organic aerosol (SOA) comprises the majority of submicron particles and is important for air pollution, health, and climate. When SOA mixes with inorganic particles containing transition metals (e.g., Fe), chemical reactions altering physicochemical properties can occur. Here, we study Fe’s impact on the formation and chemical composition of SOA formed via dark α-pinene ozonolysis on either (NH_4_)_2_SO_4_ or Fe-containing (NH_4_)_2_SO_4_ seed particles and aged at varying relative humidities (RHs). Aerosol composition was determined using online extractive electrospray ionization mass spectrometry, providing high-resolution chemical and temporal identification of monomers and dimers in the SOA. At high RH, Fe’s presence resulted in higher particulate SOA mass concentrations (117 ± 14 μg m^–3^) than those formed in its absence (70 ± 1 μg m^–3^). Enhanced mass is coupled with more dimers (C_15–20_’s), attributed to Fenton-driven oligomerization reactions. Experiments with Fe^3+^-containing seeds showed similar chemical composition and enhanced SOA mass, suggesting a dark reduction pathway to form Fe^2+^ in the presence of SOA. Overall, Fe’s presence at high RH lowers SOA volatility and enhances particulate organic mass and condensed phased reactions of higher volatility species that would normally not participate in SOA formation, which may be important when considering its formation in air quality and climate models.

## Abstract 2

Presence of Fe affects the formation of α-pinene secondary organic aerosol at high RH, through rapid Fenton-drivenoligomerization reactions, increasing gas-to-particle partitioning, andgenerating aerosol with higher mass and lower volatility.

## Introduction

Submicron atmospheric aerosols play a key role in air pollution, human health and climate.^1,2^ These aerosols consist of inorganic and organic material. For the latter, secondary organic aerosol (SOA) often makes up the majority of submicron particles by mass.^3^ SOA largely forms from oxidation of volatile organic compounds (VOCs) with various oxidants (e.g., OH O_3_, or NO_3_).^4^ This leads to reaction products with lower saturation vapor pressures, resulting in gas-to-particle partitioning and formation of SOA. These oxidation products include highly oxygenated molecules (HOMs), which form through an auto-oxidation mechanism and result in molecules with multiple hydroperoxide functional groups, and are important contributors to SOA formation.^5−7^ In the atmosphere, biogenic VOCs, and in particular monoterpenes (C_10_H_16_), are a dominant source of HOMs and SOA.^3,8,9^ Of these, α-pinene, the most globally abundant monoterpene, is responsible for a large fraction of SOA mass.^10^

In the atmosphere aerosol particles mostly exists as internal mixtures.^11^ Single particle analysis has often found SOA to be internally mixed with inorganic material such as secondary inorganic salts, e.g., ammonium sulfate ((NH_4_)_2_SO_4_) or mineral dust.^12−15^ Mineral dust often contains transition metals such as Fe.^14,16^ For example, Moffet analyzed ∼ 5000 individual particles in an air mass originating from Asian outflow and found that 5% of the particles contained Fe, accounting for an overall mass weighted fraction of Fe^2+^ of 0.33 ± 0.08.^17^ In the presence of organic acids or SO_4_^2–^, such internal mixing can lead to acid or ligand-assisted dissolution of Fe-containing minerals^18−20^ increasing the availability of soluble Fe in the aerosol particles.

In its soluble form, Fe can cycle between Fe^2+^ and Fe^3+^, termed Fenton chemistry.^21^ In the presence of organics, this can be driven by the oxidation of Fe^2+^ by peroxides (e.g., from HOMs),^22^ forming reactive oxygen species (ROS), like OH and RO radicals:^23^
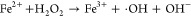
1
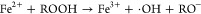
2
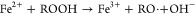
3
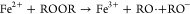
4

Contrarily, reduction of Fe^3+^ to Fe^2+^ is primarily driven through photochemical processes initiated by UV/visible light.^21^ However, in the absence of light Fe^3+^ may also be reduced to Fe^2+^ via the following reactions:^21,24 −27^
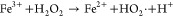
5
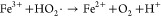
6
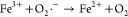
7
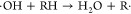
8
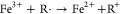
9

Aside from Fenton reactions, H_2_O_2_ and HO_2_ can also originate from particle phase HO_x_ chemistry^28−31^ and decomposition of SOA which can generate OH.^24^ As such, internally mixed aerosols containing SOA and soluble Fe provide a reactive environment, where redox reactions catalyzed by transition metals can alter the chemical composition of the SOA, and thereby their physicochemical properties.^32−36^ Given the chemical complexity of secondary organic aerosols^37−39^ and resulting variety of reactions with Fe (including its well-known complexation with species such as phenolics)^40^ considerable uncertainties still exist with how the presence of transition metals such as Fe internally mixed with SOA will alter the SOA, and hence their impact on air quality, human health and climate.^37−39^

Here, we investigate how the presence of Fe impacts the molecular composition of α-pinene SOA generated by dark ozonolysis by comparing SOA formed on (NH_4_)_2_SO_4_ and Fe-containing seed particles, respectively. Online mass spectrometry was used to gain high chemical and temporal resolution molecular data on SOA composition during aerosol formation and aging in the absence of light. SOA mass concentrations formed in the chamber were on average 70–117 μg m^–3^, which are higher than typical ambient values, but are used to generate sufficient material and to ensure that α-pinene is fully reacted away. We demonstrate that Fe impacts the fraction of monomers and dimers formed in the SOA and enhances total particulate organic mass. Furthermore, we show that these effects are strongly impacted by humidity.

## Materials and Methods

### SOA Formation and Aging Experiments

Experiments were carried out in an atmospheric simulation chamber at the Paul Scherrer Institute, Switzerland.^41,42^ Briefly, the chamber consists of a ∼8 m^3^ fluorinated ethylene propylene bag, housed in a temperature-controlled container maintained at 18 ± 1 °C. Prior to each experiment the chamber was cleaned by flushing overnight with dry, VOC and NO_x_ free air at 50 L min^–1^ from an air generator (737-250 series, AADCO Instruments, Inc., USA). This resulted in aerosol number concentrations < 10 particles cm^–3^, as measured by a scanning mobility particle sizer (SMPS model 3938; TSI Incorporated, USA). For experiments, the chamber was then filled with either dry or humidified air to achieve “high” or “low” relative humidity conditions (RH). Humidified air was generated by passing 20–30 L min^–1^ of air through a 2 L heated round-bottom flask filled with 18.2 MΩ cm Milli-Q water, while chamber RH was monitored using a probe (Vaisala, model HMP 110). For “high RH” experiments, the chamber was preconditioned to 90–95% RH, which ensured aerosol remained deliquesced throughout the experiments. For “low RH” experiments, the chamber remained below 10% RH. Once the chamber had reached the desired RH, ∼300 parts-per-billion-by-volume (ppbv) O_3_ was added. O_3_ was generated by passing dry air through a UV lamp (λ = 254 nm), and the concentration in the chamber was continuously monitored by a UV-absorption monitor (Thermo Environmental Instruments, TEI 49C).

Seed particles were then directly nebulized into the chamber, until a seed aerosol mass concentration of 72 ± 8 μg m^–3^ (assuming a density of 1.2 g cm^–3^) was reached. In reality, the density likely differs since the seed particles are a mixture of (NH_4_)_2_SO_4_ and FeSO_4_ and as such have a density between 1.8 g cm^–3^ (for FeSO_4_ heptahydrate) and 2.7 g cm^–3^ (anhydrous FeSO_4_). These densities were calculated based on the average density for a 50:50 mixture of (NH_4_)_2_SO_4_ and the respective Fe salt, and would result in seed particle masses between ∼105–160 μg m^–3^. Furthermore, the reported aerosol mass concentrations represent dry particle mass, since a Nafion drier was used upstream of the SMPS. (NH_4_)_2_SO_4_ (AS) seed particles were generated using a solution of ∼3 g L^–1^ (NH_4_)_2_SO_4_ (Sigma-Aldrich, ≥ 99%) in 18.2 MΩ cm Milli-Q water. Fe-containing (Fe^2+^/AS or Fe^3+^/AS) seed particles were generated using a solution containing equivolumes of ∼5 g L^–1^ iron (II) sulfate (Fluka Chemika, > 99.5%) or ∼3 g L^–1^ iron (III) sulfate (Alfa Aesar, Reagent grade) in 18.2 MΩ cm Milli-Q water mixed together with the (NH_4_)_2_SO_4_ solution described above. Solutions containing Fe were prepared immediately before the experiments and covered in foil to minimize light exposure and potential photochemical and oxidation reactions. α-pinene (3.2 μL; TCI, > 97.0%) was then injected using a syringe through a heated (80 °C) septum, and flushed (∼60 L min^–1^) into the chamber, resulting in a mixing ratio of ∼50 ppbv. Although the absolute mixing ratios are higher than typical ambient concentrations, the aim of our experiments was to generate SOA that is compositionally similar to ambient aerosol. The α-pinene:O_3_ ratios used to form the SOA are similar to conditions found in the Finnish Boreal forest.^43,44^ Furthermore, the O_3_ mixing ratios are at a maximum an order of magnitude higher than those found in the atmosphere, since O_3_ concentrations often exceed 100 ppbv in urban environments.^45^ Following α-pinene addition, all input flows were stopped and the chamber was operated in batch mode for the remainder of the experiment. Hence, the chamber volume slowly decreased as air was sampled from the connected instruments. A summary of experiments conducted for the different RH and seed cases can be found in Table S1.

### Aerosol Measurements

Throughout the experiments, the dry particle size distribution was monitored using a SMPS. The chemical composition of SOA was measured online with an extractive electrospray ionization time-of-flight mass spectrometer (EESI-ToF, Tofwerk), which has been described elsewhere.^46,47^ It consists of a home-built EESI inlet^46^ coupled to an atmospheric pressure interface time-of-flight mass spectrometer (APi-ToF; Tofwerk AG) with a resolution of ∼5000 Δ*M*/M. Aerosol was sampled at a flow rate of ∼0.8 L min^–1^ through an extruded carbon denuder into a plume of charged droplets generated by the electrospray probe (operated between 2700 and 3000 V) from a solution containing 100 parts-per-million (ppm) NaI in 18.2 MΩ cm Milli-Q water. Positive ion mass spectra were recorded at 1 Hz, and all ions were detected as adducts with Na^+^.

### EESI-ToF Data Analysis

Analysis of EESI-ToF data was performed using Tofware version 3.2.5 (Tofwerk AG, Thun, Switzerland). One Hz raw data were averaged to 10 s before high-resolution peak fitting was performed for *m*/*z* 95 – *m*/*z* 535. Fitted peaks were assigned molecular formulas with carbon numbers ranging from C_2_ to C_20_. Background measurements were conducted every 6.5 min (i.e., 5 min of chamber air followed by 90 s of background), by sampling chamber air through a HEPA filter. Reported signals were determined as the difference between sample and the average background immediately before and after each measurement period. Data were then averaged to 6.5 min. Additionally, data were normalized to the primary ion, Na_2_I^+^. EESI-ToF signals are reported as an ion mass flux (attograms per second; ag s^–1^) reaching the mass spectrometer microchannel detector. To determine the mass flux, the measured EESI-ToF signal (in ion counts s^–1^) was converted on a per ion basis using the following formula:
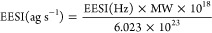
10

The SMPS and EESI-ToF data were not corrected for losses of aerosol to the chamber walls since this work focused on the intercomparison to individual experimental time points.

## Results and Discussion

### Impact of Fe on SOA Mass Yields

**Figure 1:**
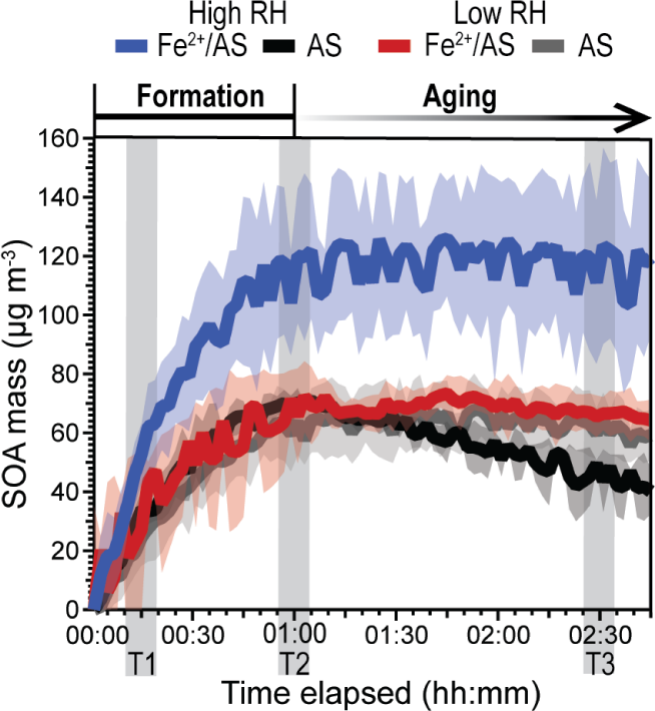
Time series of average aerosol mass (in μg m^–3^) generated from the dark ozonolysis of α-pinene (using ∼300 ppb O_3_ and ∼50 ppb α-pinene) in the presence of different seed particle types (with and without Fe) at high and low RH. Variation in the replicate experiments is shown as ± 1 standard deviation (shaded region). Gray shaded regions labeled T1, T2 and T3 represent case study periods. A summary of SOA mass formed for all experiments is in Table S1 and summary of SMPS aerosol mass data in Figure S2.

Figure 1 shows time series of the average SOA mass concentration generated via dark ozonolysis of α-pinene in the presence of different seed particle types, at low (RH < 10%) and high (RH > 80%) humidities. Maximum SOA mass concentrations for all experiments were observed ∼1 h after α-pinene addition, which was consistent with gas-phase depletion of α-pinene as simulated using the F0am model (see Figure S1). At low RH, all experiments (independent of seed type) generated a maximum SOA mass of ∼65 μg m^–3^ (67 ± 8 μg m^–3^ for Fe^2+^/AS seed and 62 ± 5 μg m^–3^ for AS seed). The SOA mass concentration was determined by subtracting the seed from total aerosol mass concentration measured by the SMPS. When AS seeds were used at both low and high RH conditions they had a similar SOA mass concentration (70 ± 1 μg m^–3^). By contrast, in the presence of Fe^2+^/AS seeds and at high RH, a maximum SOA mass of 117 ± 14 μg m^–3^ formed. This demonstrates that Fe can enhance SOA formation. Note that every experiment has been repeated at least 3 times and the standard deviations shown as shaded regions indicate the consistent effect of Fe on SOA formation at high RH. To explore the effect of Fe on the composition of SOA during both formation and its evolution, 3 periods (marked T1, T2 and T3 in Figure 1) were selected for detailed chemical analysis.

### Chemical Composition of SOA Formed in the Presence of Fe-Containing Seeds

**Figure 2:**
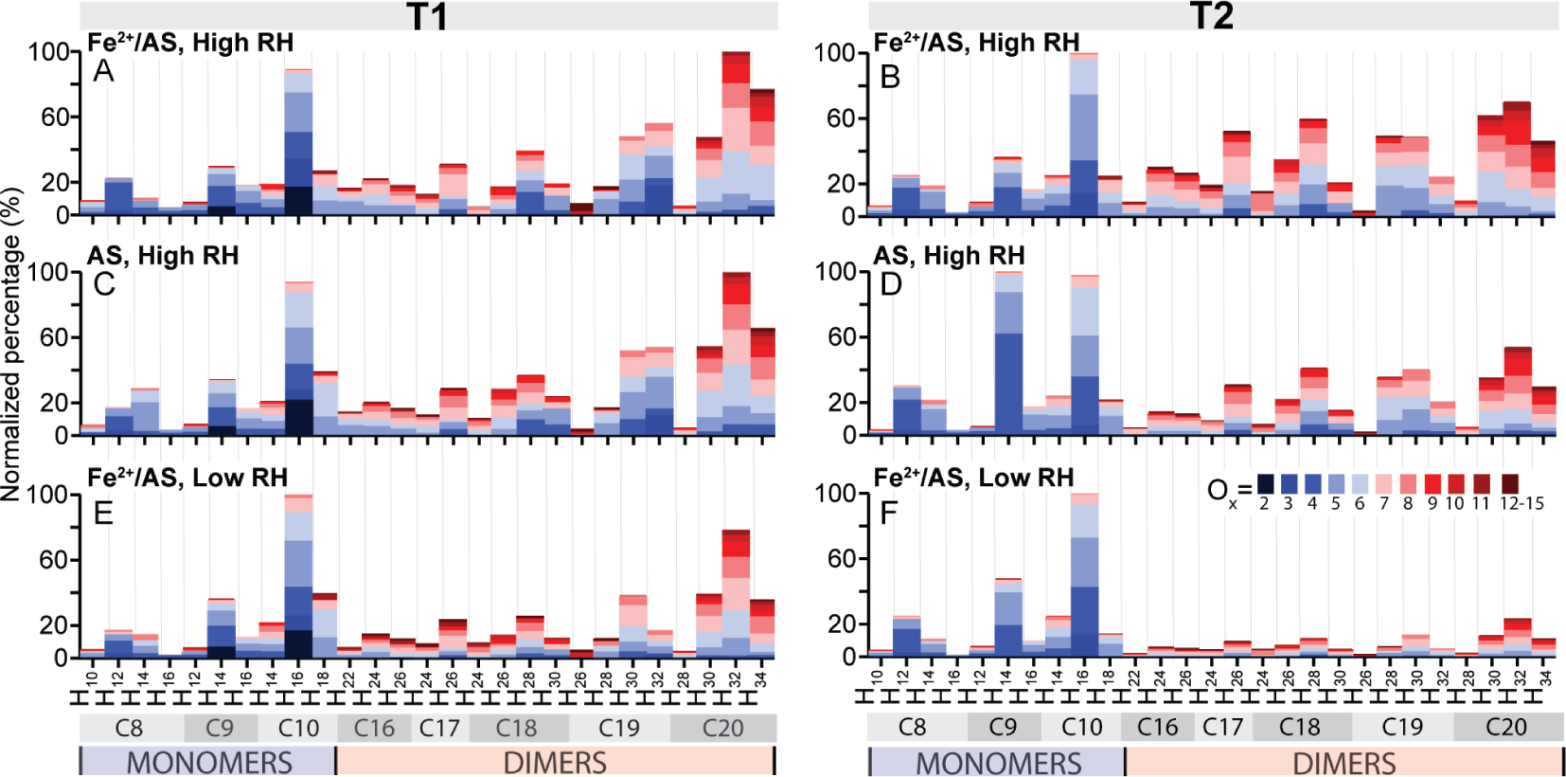
Bar graphs of key monomers and dimers for experiments conducted at high RH on (A/B) Fe^2+^/AS and (C/D) AS seed particles, and at low RH on (E/F) Fe^2+^/AS seed particles. A, C and E depict the average composition of aerosol ∼15 min after the addition of α-pinene (T1, Figure 1), and B, D and F show the average composition of aerosol ∼1 h after the addition of α-pinene (T2, Figure 1). Data are shown as a normalized percentage relative to the most abundant summed species (ΣC_x_H_y_O_z_) at the time depicted.

The chemical composition at T1 and T2 are summarized in Figure 2. We show the data as average chemical composition, in terms of the key monomers (C_8–10_) and dimers (C_16–20_), respectively, for the different experimental conditions. Replicate measurements for a given experimental condition showed strong correlation (*R* > 0.8, Figure S3).

At T1, the abundance of monomers and dimers was similar for all our experimental cases. Monomers and dimers were dominated by C_9,10_ and C_19,20_ species, respectively. The composition during the early stage of SOA formation is consistent with condensation and gas-to-particle partitioning of low and semivolatile oxidation products from the reaction of OH and O_3_ with α-pinene, since an OH scavenger was not used.^48−51^ Specifically, C_10_H_16_O_2–6_ monomers were abundant in all cases, as indicated by the respective bars, where the number of oxygen (O_x_) is denoted as different colors. At T1, all experimental cases also showed a high fraction of dimers, with C_20_H_30–34_O_x_ being the most abundant species. C_20_’s are known to form in the gas-phase from RO_2_ + RO_2_ recombination reactions and will rapidly condense onto the preexisting aerosol because of their extremely low volatility.^51^ For all experimental cases, the C_20_ dimers contributed on average, the largest fraction to the carbon distribution at T1.

Despite these similarities among all experimental cases at T1, several distinct differences were observed for experiments with Fe^2+^/AS seeds at high RH (Figure 2A). One noted difference was the fraction of C_10_H_18_O_x_ monomers, which was greater for the low RH Fe^2+^/AS (39%) and high RH AS cases (39%) compared to the high RH Fe^2+^/AS case (27%). Unlike C_10_H_16_O_x_, which form through either O_3_ or OH pathways, C_10_H_18_O_x_ monomers mostly form as products from OH chemistry,^51,52^ and have been shown to rapidly decay in α-pinene SOA, suggesting they are reactive, peroxide containing molecules.^42^ Known peroxides, such as C_10_H_18_O_4_, can also form through the reaction of the Criegee intermediate (C_10_H_16_O_3_) with H_2_O,^52^ which has been found as an important termination pathway at high RH.^53,54^ Hence, their presence is expected especially during the high RH experiments. However, the abundance of peroxide functionalization in C_10_H_18_O_x_ molecules also makes them prone to participate in reactions with Fe^2+^ via Fenton chemistry (e.g., R4), resulting in the observed lower C_10_H_18_O_x_ content in the presence of Fe (Figure 2A).

At T2, the composition for different experimental conditions diverges. For example, for the high RH AS seed case, monomeric C_9_H_14_O_x_ molecules became a dominant aerosol component. By contrast, both RH cases with Fe^2+^/AS seeds were dominated mostly by C_10_H_16_O_x_ monomers. The fraction of dimers was higher for experimental conditions at high RH (60% - Fe^2+^/AS, 49% - AS), when compared to low RH (35%). Further, the C_16–18_ molecules exhibit clear differences when Fe was present at high RH: their relative abundances increased from T1 to T2 (23% to 30%) for the high RH Fe^2+^/AS case, versus staying roughly constant (23% to 21%) at high RH AS, or slightly decreasing for low RH Fe^2+^/AS (20% to 14%). These results demonstrate that Fe plays a substantial role in the formation of these dimers. Given the similarity between the Fe^2+^/AS and AS seeds under low RH conditions (Figure S1, *R* > 0.95), there are limited interactions between Fe and α-pinene SOA under low RH conditions. Under high RH conditions with deliquesced particles, Fe and α-pinene SOA likely mixes better and results in the composition deviations compared to low RH conditions in Figure 2.

### Temporal Evolution of SOA Monomer and Dimer Composition in the Presence of Fe

**Figure 3:**
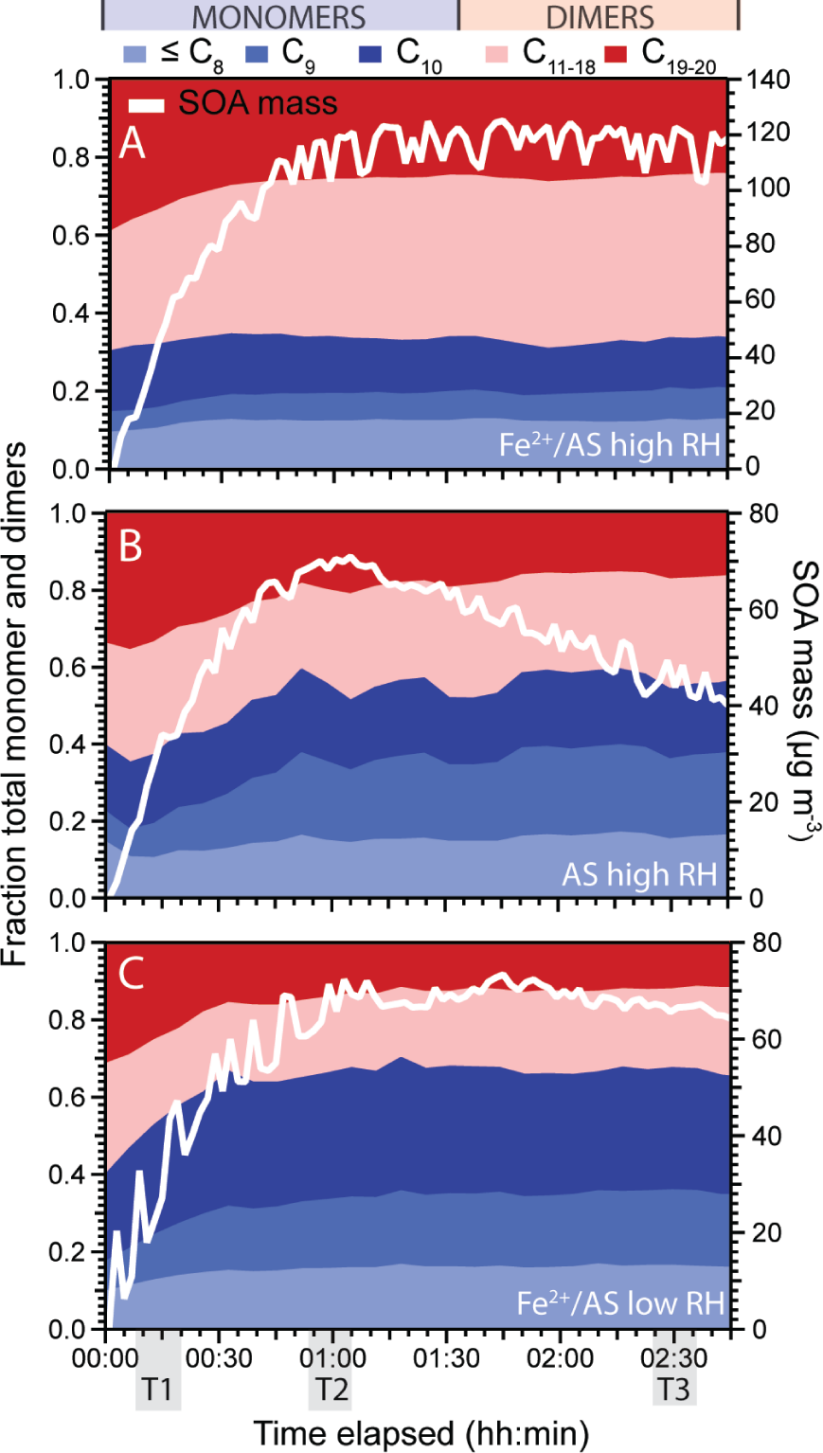
Time series for monomers (blue) and dimers (red) for different experimental cases: (A) high RH Fe^2+^/AS, (B) high RH AS and (C) low RH Fe^2+^/AS. Monomers and dimers are shown as a fraction of the total mass flux. The average SOA mass concentration is also shown as the white trace. Normalized time series for the monomers and dimers shown here, are in Figure S4.

Complementary to the detailed SOA composition data for T1 and T2 described above, Figure 3 shows the trend in monomers and dimers for each seed and RH case over the entire experiment. For all cases, the dimer fraction was greatest at the beginning of the experiments, and represented 60–70% of total signal in the first 5–10 min of SOA formation. This is due to the rapid gas-phase formation and condensation of dimers onto the preexisting particles due to their extremely low volatility, building up an organic phase for the other organics to partition into. However, for the high RH AS and low RH Fe^2+^/AS cases, a steady decrease in the contribution of dimers compared to monomer until maximum SOA mass (at T2) occurred. This gradual decrease in dimers (from 60% to 49% for the high RH AS, and from 60% to 32% for the low RH Fe^2+^/AS) is likely due to the gas-to-particle partitioning of more volatile oxidation products as SOA forms.

In contrast, the ratio of dimers to monomers remained relatively consistent at around 70% for the high RH Fe^2+^/AS seed case (Figure 3A). This nearly constant relative abundance of monomers to dimers suggests rapid condensed-phase dimer formation catalyzed by Fe. As monomers react to form dimers, the monomers will be’consumed’ in the aerosol phase resulting in further gas-to-particle partitioning of semi- and low volatility species to maintain equilibrium with the particle phase. This reactive uptake of monomers leads to the increase in overall aerosol mass observed at high RH (white trace in Figure 3). Likewise, the monomers in the aerosol phase will continuously oligomerize as long as there is sufficient interaction with soluble Fe. As such, Fe-catalyzed particle-phase monomer to dimer conversion will create a spiraling increase in SOA mass until all the gas-phase reactants are consumed, and a steady state is reached, i.e., at maximum aerosol mass. When all α-pinene has been consumed the gas-phase monomers have two fates, either reactive absorptive partitioning into the particle phase or absorptive loss to the walls of the chamber. Therefore, after the α-pinene has been fully consumed, the limit to the increase in the aerosol mass will be the competition between the walls and the rate of the reaction in the aerosol phase. Considering the aerosol mass increase slows at 1hr, most changes occurring from T2 to T3 are likely coming from other aging reactions in the particle-phase that slowly alter the particle-phase composition.

Furthermore, this condensed-phase monomer to dimer conversion is evident through a comparison of Figure 3 with the dry aerosol mass data at T2 (Figure 1). In both high RH cases, monomers represented ∼35 μg m^–3^ aerosol, despite total SOA mass being nearly double in the presence of Fe. Dimers represented ∼85 μg m^–3^ for Fe^2+^/AS experiments and only ∼25 μg m^–3^ for AS experiments, under high RH conditions, assuming similar instrument sensitivities between the SMPS and the EESI-ToF. However, since the EESI-ToF sensitivity toward dimers is known to be lower than to semivolatile monomers,^55^ these values likely represent lower limit estimates. For example, if the sensitivity estimates from Bell et al.^55^ are applied for high RH experiments the contribution of monomers would decrease to ∼26 μg m^–3^ and would increase to ∼95 μg m^–3^ for dimers for Fe^2+^/AS experiments. For the AS experiments the monomers would decrease to ∼22 μg m^–3^ and dimer increase to ∼38 μg m^–3^, respectively. As such, SOA formed at high RH in the presence of Fe likely comprises significantly more dimers compared to SOA formed in the absence of Fe.

### Impact of Fe on the Lifetime of SOA Dimers

**Figure 4:**
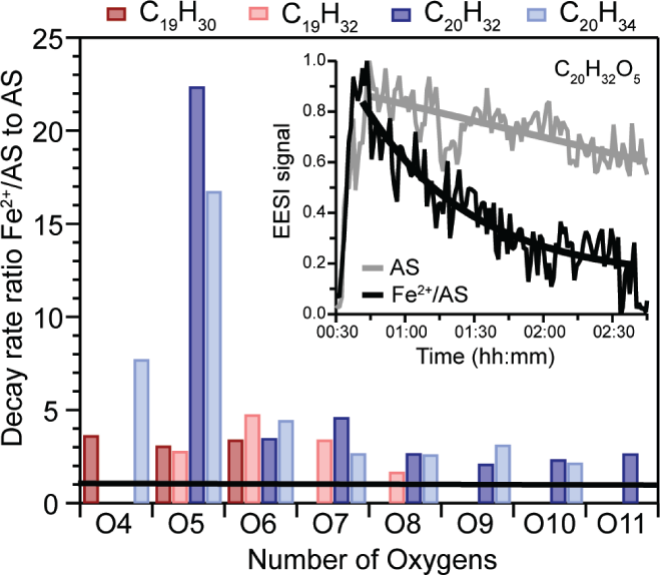
Ratio between decay fits for C_19_ (red bars) and C_20_ (blue bars) dimers vs their number of oxygen (*x*-axis) for Fe^2+^/AS and AS high RH experiments. The black bar represents the self-normalized 1:1 ratio for AS experiments, i.e., points falling above this line represent dimers that decayed faster in the presence of Fe, than in its absence. Insert: Time series of C_20_H_32_O_5_ at high RH in the presence and absence of Fe. The exponential fitting functions applied to these data can be seen as the overlaid trace with the same color.

To further explore how the presence and absence of Fe mediates the temporal evolution of condensed phase products at high RH, time series for individual monomers and dimers were analyzed for this experimental case (Figure S4). Analysis revealed a distinct decay of C_19–20_ dimers, which is exemplified in Figure 4 (insert), by the time series for C_20_H_32_O_5_, whose decay was particularly prominent. In this example, the EESI-ToF signal intensity for C_20_H_32_O_5_ decayed notably faster for the Fe^2+^/AS, than for the AS seed case. To explore dimer lifetime more quantitatively, decay rates were derived by fitting the individual time series with an exponential fit:
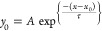
11where y_0_ is the fitted 1-min averaged time series, A is the initial y-value (at EESI-ToF signal maximum), x is the time value at steady state, x_0_ is a constant for the initial time value and τ is the lifetime in seconds. A summary of the decay fits for species that exhibited a different decay trend in the presence of Fe is given in Tables S2–3. Overall, the decay rates for more saturated C_19_H_30–32_ and C_20_H_32–34_ dimers were on the order of ∼10^–4^ s^–1^ to ∼10^–3^ s^–1^ for high RH AS and Fe^2+^/AS seed cases, respectively.

To better illustrate the role of Fe, Figure 4 shows the ratio in the decay rates between Fe^2+^/AS and AS seed cases, for C_19_ (red) and C_20_ dimers (blue) of variable oxygen content. Most dimer species exhibited decay rates enhanced by a factor less than 5 in the presence of Fe. However, certain species such as C_20_H_32_O_5_ and C_20_H_34_O_5_’s decayed ∼16 and 22 times faster in the presence of Fe at high RH. Overall, the faster decaying species mainly form from RO_2_ + RO_2_ reactions^48,51^ and as such are expected to contain a peroxide linkage, which will be prone to degradation via R4. Furthermore, this increased decay was not observed for less saturated C_19_H_26–28_ and C_20_H_28–30_ dimers, which form primarily through O_3_ reactions, presumably because they contain fewer peroxide functional groups than dimers with a higher degree of saturation, i.e., C_19_H_30–32_ and C_20_H_32–34_’s. As such, these dimers may more readily participate in Fenton reactions in the condensed-phase. Additionally, it is important to note that the aerosol system is in perpetual evolution especially at high RH and in the presence of Fe. As shown in Figure 3, a gradual increase in C_11–18_ dimers was observed as aerosol aged, perhaps from the degradation of C_20_’s; although due to the complexity of the system it remains unclear whether this occurs as a direct result of their decay or indirectly. In contrast, this trend was not observed under low RH conditions (Figure S5), which may indicate limitations of this chemistry due to aerosol mixing state.

### Impact of Fe on SOA Aging

**Figure 5:**
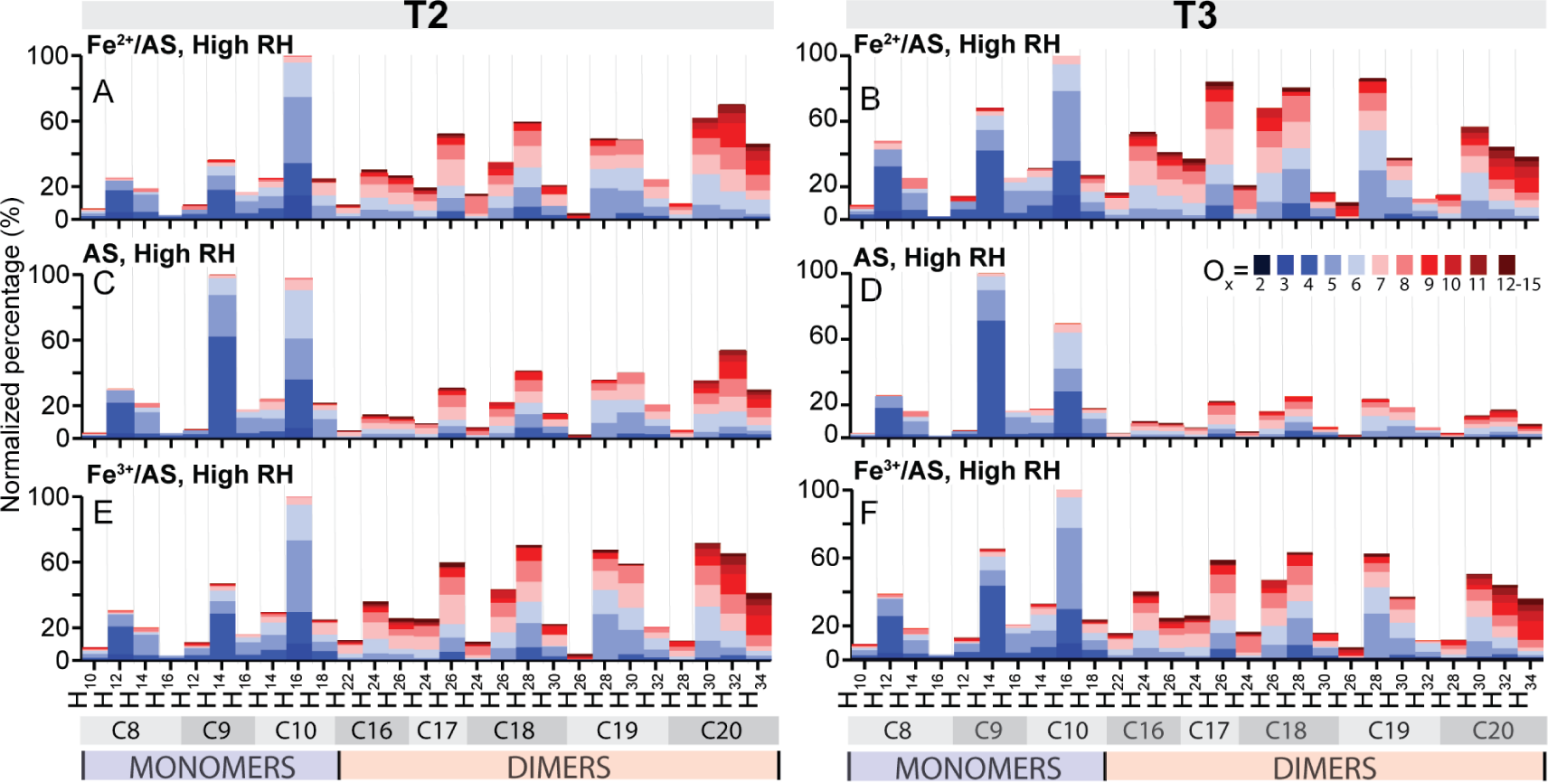
Bar graphs of key monomers and dimers for experiments conducted at high RH on (A/B) Fe^2+^/AS (C/D) AS, and (E/F) Fe^3+^/AS seed particles. A, C and E depict the composition of aerosol ∼1 h after the addition of α-pinene (T2, Figure 1), and B, D and F show the composition of aerosol at ∼2.5 h (T3, Figure 1).

The longer term (aging) effects of Fe on SOA were explored by comparing changes in aerosol composition at T2 and T3. The composition at these two periods is shown in Figure 5, and summarized in Figure S6 as percent difference plots. A general increase from T2 to T3 in monomers and dimers for the high RH Fe^2+^/AS seed case (Figure 5A,B) was noted, in particular for lower carbon number dimers, i.e., C_16–18_’s (30% to 37%), and C_8–9_ monomers (13% to 17%). This is consistent with continued fragmentation chemistry in the presence of Fe at high RH. In addition to fragmentation, there was also a lower degree of saturation, compared to α-pinene SOA aged in the absence of Fe (Figure 5C,D) (e.g., transformation from C_20_H_32_O_x_ to C_20_H_30_O_x_ molecules). In contrast, for low RH Fe^2+^/AS experiments (Figure S7), comparatively few changes arose. This is consistent with limited interactions between Fe and α-pinene SOA, likely resulting from limited mixing, as discussed above. The continued transformation via fragmentation reactions and decrease in the degree of unsaturation demonstrates that Fe, when well mixed within the SOA, continually alters the chemical composition of α-pinene SOA well after the maximum SOA mass is reached (and the α-pinene is fully consumed).

Such continued changes in SOA chemical composition, as observed here, also require that Fe remains available in reactive form. Thus, efficient recycling from Fe^3+^ back to Fe^2+^ must occur, as Fe^2+^ is expected to be consumed rapidly during the initial gas-to-particle partitioning of the peroxide species at around T1. To test this hypothesis, we performed further experiments where instead of Fe^2+^/AS seeds, we used Fe^3+^/AS seeds (see SI), otherwise following the same experimental procedure. The composition of SOA formed on Fe^2+^/AS seeds, discussed so far, to that of SOA formed on Fe^3+^/AS seeds is also shown in Figure 5. Comparing the results of panels A/B and E/F in Figure 5 reveals that both types of Fe-seeds led to very similar SOA composition (*R* > 0.8, Figure S1). This suggests that a dark reduction pathway for Fe^3+^ must exist already early on to facilitate production of Fe^2+^, and Fenton reactions R5–9. This Fe-recycling chemistry will be described in more detail in the following section.

### Possible Implications of the Presence of Fe on SOA Chemistry in the Atmosphere

Our results showcase the importance of Fe on mediating the chemical composition of SOA (during both formation and aging) formed via dark ozonolysis of α-pinene, when Fe-containing seed particles are internally mixed with the SOA phase at high RH. Specifically, the presence of Fe was demonstrated to increase dimer formation and SOA mass, increase fragmentation reactions, and decrease the lifetimes of saturated C_19–20_ dimers.

Fenton chemistry through reactions with Fe^2+^, will result in the formation of OH radicals by R1–2, and RO radicals from ROOH and ROOR functional groups via R3–4. OH radicals will propagate radical chemistry through hydrogen abstraction reactions generating carbon centered radicals, which can form RO_2_ radicals via uptake of O_2_. RO radicals can proceed down similar pathways, by unimolecular rearrangement reactions through hydrogen abstraction and ring-opening reactions, or by undergoing fragmentation reactions,^51^ previously hypothesized in gas-phase chemistry. Though reaction with O_3_ is expected to form mostly carbonyl and peroxide functional groups, reactions with Fe^2+^ can convert peroxides to alcohols, which could lead to the formation of hemiacetals or dimers.^56^ Additionally, the presence of Fe^2+^ will result in high concentrations of both carbon centered radicals (via reactions with OH), RO radicals, and RO_2_ radicals, all of which could undergo radical recombination reactions to form dimers. Both of these pathways (radicals or alcohol formation) can promote formation of dimers in the presence of Fe and suggests that Fenton chemistry, even in the absence of light, is an effective means to promote formation of higher order oligomers and lower volatility components. The presence of Fe could also in theory act as a catalyst promoting the formation of dimers, without needing to invoke the role of Fenton chemistry. Although we cannot conclusively elucidate the exact pathway of their formation with the current experiments, the overall implication of the presence of Fe is the promotion of oligomers. The results herein demonstrate that at high RH when Fe is presumably well-mixed, the dimer fraction is nearly constant despite a significant increase in (organic) particulate mass (i.e., Figure 3A), meaning there is rapid conversion of SVOCs partitioning to the aerosol into oligomers, and creation of SOA that would not otherwise form a significant fraction of particulate SOA.

When Fe^2+^ is consumed, the mechanism for OH and RO radical formation and thereby fragmentation reactions, in theory, should be slowed. The maintained production of C_16–18_ dimers and C_8–9_ monomers, suggests that a dark, i.e., nonphotochemical pathway must exist for the dark reduction of Fe^3+^ to Fe^2+^, in order to sustain this chemistry, if Fenton chemistry is indeed responsible. This is supported in Figure 5E,F where aerosol composition at T2 and T3 for Fe^3+^/AS experiments are shown. As with Fe^2+^/AS seed experiments, the Fe^3+^/AS had a larger fraction of dimers compared to monomers, that was maintained as aerosol aged in the dark. Since the experiments started with Fe^3+^ instead of Fe^2+^, certainly, a dark reduction pathway must exist to reduce Fe^3+^ to Fe^2+^. We hypothesize that this could occur via reaction with HO_2_ (R6) or a carbon-centered radical (R9), which may be enhanced in the presence of Fe due to increased OH initiated H-abstraction reactions (R8). However, the exact mechanism through which this occurs remains speculative at present and requires future experiments to elucidate. Moreover, these reactions can act as a source of H_2_O_2_ and HO_2_ in the particle phase,^21^ further reducing Fe^3+^ back to Fe^2+^ and maintaining the reaction cycle and production of OH, although, likely at a slower rate, compared to the oxidation reactions. For example, comparison of rate constants for bulk reactions for H_2_O_2_ are ∼6 orders of magnitude slower for Fe^3+^ (reduction) than for Fe^2+^ (oxidation).^57^ Additionally, although the decomposition of SOA in the presence of Fe^2+^ has been shown to generate OH,^24^ the decomposition of organic peroxides and carbonyls in the absence of Fe can generate a variety of radicals (e.g., OH radical, HO_2_ radical, organic radicals R/RO, etc.) in SOA^58^ potentially providing a non-Fenton pathway for the reduction of Fe^3+^. Such Fe recycling also means that the dark Fenton chemistry is catalytic with respect to Fe and suggests that Fe likely has an impact on SOA aging also at lower Fe concentrations than tested here (∼100 mM). Furthermore, under photo-Fenton conditions Fe^3+^ recycling would likely be enhanced. As such, the full impact of Fe on SOA, including to which degree fragmentation and oligomerization reactions occur, requires further elucidation. In particular, these studies should be done at Fe concentration more similar to those typically found in ambient aerosol (0.001–1 mM Fe^3+^ in cloud droplets and aerosol particles in rural and urban environments).^36^ As well as, at longer aging periods, since well mixed aerosol with lower Fe concentrations may still exhibit similar changes in composition over extended periods due to Fe recycling.

Further studies should also include detailed gas-phase measurements (e.g., of HOMs) to help decouple changes in SOA composition that are driven by gas-to-particle partitioning vs condensed phase oligomerization and fragmentation reactions, which were beyond the scope of the work presented here. Specifically, studies should explore the fate of products formed via OH oxidation. These products were shown to be in lower relative abundance during initial SOA formation compared to O_3_ oxidation products, i.e., the lower relative signal of C_10_H_18_ monomers observed in Figure 2A compared to other experimental conditions. As such, they are likely to contain highly reactive functional groups such as peroxides making them prone to react with Fe in aerosols. Additionally, the role of oxidants such as dissolved O_2_, should be explored to determine the impact they have on formation of radicals, e.g., RO_2_, in the condensed phase, especially under high RH conditions when diffusion is not expected to be limited. Although O_3_ represents one of the main oxidants in our experiments and could result in the oxidation of Fe^2+^, we do not expect it to participate in further condensed phase reactions with organic species since this would require the presence of a carbon double bond which would not remain following initial gas-phase oxidation. Finally, the role of aerosol pH should be explored in more detail, especially at very low pHs where acid catalyzed oligomerization reactions could become an important and potentially competing pathway for dimer formation.^59,60^

At the same time, our results demonstrate that the presence of Fe alone is insufficient to drive this chemistry, as we only observed considerable changes in SOA composition at high, but not at low RH. It is well established that RH affects the microphysical properties of organic–inorganic mixtures, as studied here, including their phase state^61−63^ hygroscopic growth and mixing behavior^11^ with feedbacks for reactivity.^62^ As all these properties are known to be a strong function of RH, future experiments should focus on the intermediate RH range not explored herein. Although experiments conducted here were conducted at either high or low RH to demonstrate the two extremes, preliminary experiments conducted at ∼50% RH (Figure S12) show significant dimer formation occurs at lower RH and demonstrating that the chemistry discussed herein can be applied to a broader range of ambient RHs. Additionally, studies have shown that ambient RH at 25 °C is frequently above 75% RH,^65^ based on these parameters the aerosol particles are expected to be mostly liquid. Furthermore, Shiraiwa et al.^66^ showed that near the surface over forested regions, rich with biogenic emissions, that organic diffusion time scales should be on the order of seconds.^67^ As such, our work demonstrates this chemistry can be relevant across a broad range of atmospheric RH ranges. Nevertheless, additional work is needed to quantify more precisely the RH thresholds under which Fenton chemistry effects the overall atmospheric aerosol population.

Overall, the chemical processes described herein, have considerable impacts on SOA composition and mass concentrations. As such, they should be considered when evaluating their impacts on air pollution, health and climate, especially in regions where SOA and Fe-containing inorganic particles such as mineral dust become internally mixed.

## Supporting Information Available

Summary of experiments conducted (Table S1), evaluation of α-pinene depletion using the F0am 0D model (Figure S1), time series for aerosol mass concentrations for individual experiments (Figure S2), Pearson correlation plot for replicate experiments (Figure S3), time series for monomers and dimers (Figure S4), decay rates and lifetimes for selected C_19_ and C_20_ dimers (Tables S2 and S3), decay rate ratio for low RH Fe^2+^/AS experiments (Figure S5), relative percent changes in key monomers and dimers between T2 and T3 (Figure S6), bar graph of key monomers and dimers at T3 for low RH Fe^2+^/AS case (Figure S7), summary of SMPS and EESI data for Fe^3+^/AS experiments (Figures S8–S11), bar graphs of Fe^2+^/AS experiments at different RHs (Figure S12) (PDF)
