# Supplementary material for: Iron-Containing Seed Particles Enhance α-Pinene Secondary Organic Aerosol Mass Concentration and Dimer Formation
Source: Environ Sci Technol. 2024 Sep 10;58(38):16984–93. doi: 10.1021/acs.est.4c07626 (PMC11428137; doi:10.1021/acs.est.4c07626)
Supplement: Supplementary file 1 — es4c07626_si_001.pdf [file es4c07626_si_001.pdf]

SUPPORTING INFORMATION

Iron-containing seed particles enhance α-pinene secondary organic aerosol mass concentration and dimer formation

Natasha M. Garner[1], Jens Top[1], Fabian Mahrt[1, †], Imad El Haddad[1], Markus Ammann[1], David M. Bell[1]**

1: PSI Center for Energy and Environmental Sciences, Paul Scherrer Institute, 5232 Villigen, Switzerland

†: Now at: Department of Chemistry, Aarhus University, 8000 Aarhus, Denmark

email: david.bell@psi.ch or markus.ammann@psi.ch

Research article for Environmental Science and Technology

August 23, 2024

This file contains:

Pages S1 to S20

Figures S1 to S12

Tables S1 to S3

18 References

TABLE OF CONTENTS

S1. EXPERIMENT OVERVIEW……………………………………………………………S3

S2. F0AM 0D BOX MODEL…………………………...………………...…………….S4-5

S3. SMPS TIMESERIES ……..…………………………………………...……...…….S6

S4. PEARSON CORRELATION COEFFICIENT ………………..………...…………S7-8

S5. MONOMER AND DIMER TIMESERIES………………………………...…………S9

S6. DECAY RATES……………………………………………………………….S10-12

S7. AGING OF FE CONTAINING SOA…………………………………..……………S13

S8. T3 BAR GRAPH LOW RH FE^{2+}/AS CASE…………..…….……………..…………..S14

S9. FE^{3+}/AS EXPERIMENTS……………………..…...……………..…...……..…………S15-17

S10. COMPOSITION OF FE^{2+}/AS EXPERIMENTS AT DIFFERENT RHS…………….S18

S11. REFERENCES………………………………………………………..……...S19-20

S1. EXPERIMENT OVERVIEW

Table S1. Summary of experiments conducted at low and high relative humidity (RH) with seed particles containing either ammonium sulfate (AS) or a mixture of iron (2+/3+) sulfate and ammonium sulfate (Fe^{2+}/AS or Fe^{3+}/AS).

Experiment	Seed type	Seed mass (μg m^{-3})	O_3 (ppbv)	α-pinene (μL)	SOA mass (μg m^{-3})	RH (%)	O/C (~1 h)
1	Fe^{2+}/AS	64	313	3.2	109	78	0.59
2	Fe^{2+}/AS	73	307	3.2	131	>95	0.55
3	Fe^{2+}/AS	85	255	3.2	143	>95	0.63
4	AS	74	248	3.2	70	89	0.62
5	AS	71	346	3.2	72	>95	0.61
6	AS	73	333	3.2	74	93	0.64
7	Fe^{2+}/AS	71	~300	3.2	65	<10	0.66
8	Fe^{2+}/AS	68	324	3.2	59	<10	0.67
9	Fe^{2+}/AS	65	322	3.2	53	<10	0.64
10	Fe^{2+}/AS	67	287	3.2	64	<10	0.65
11	Fe^{3+}/AS	89	283	3.2	99	>95	0.58
12	Fe^{3+}AS	71	352	3.2	88	91	0.60
13	Fe^{3+}AS	64	325	3.2	75	93	0.63

S2. F0AM 0D BOX MODEL

The depletion of gas-phase SOA precursor, α-pinene, was simulated using the F0am 0D atmospheric box model.[3] The model was run using a subset of the Master Chemical Mechanism (MCM) model (version 3.3.1)[4, 5] which included 314 species and 942 reactions. This MCM subset was generated based on non-aromatic,[4, 6] aromatic[7, 8] and β-caryophyllene[9] reaction schemes. Initial concentration inputs were set to 50 ppb α-pinene and 300 ppb O_3, to simulate chamber conditions at the beginning of all experiments. Simulations were run at both low (5%) and high (95%) RH to evaluate the depletion of α-pinene under different RH scenarios that were evaluated experimentally in this work.

Figure S1 shows the average aerosol mass (in μg m^{-3}) as determined by SMPS for the experiments, where the different aerosol seed types are shown as the colored traces. The simulated depletion of gas-phase α-pinene (in ppb), is shown as the dashed line. Both the measured aerosol mass and simulated depletion of gas-phase α-pinene exhibited similar trends; peak aerosol mass and full depletion of α-pinene occurred around ~1 h after the injection of α-pinene into the chamber at 00:00 h.

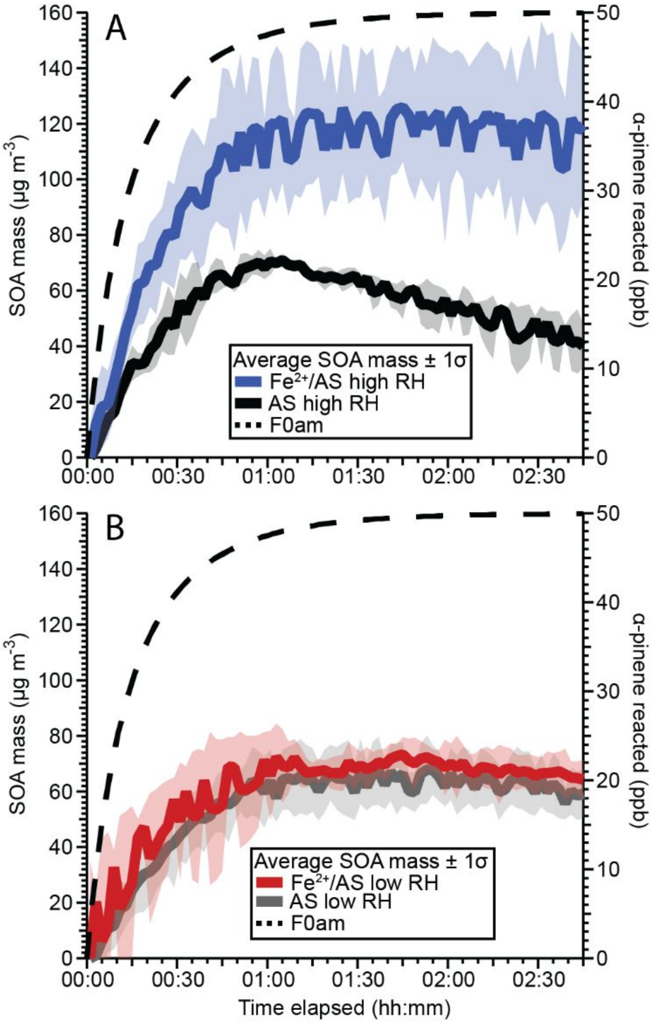

Figure S1. Comparison of average SOA mass ± 1 standard deviation (in µg m^{-3}) formed from the dark ozonolysis of α-pinene SOA in the presence of AS or Fe^{2+}/AS seed particles, with the modeled depletion of α-pinene (in ppb) as calculated by the F0am 0D model. Figure S1 shows simulations conducted at (A) 95% and (B) 5% RH.

S3. SMPS TIMESERIES

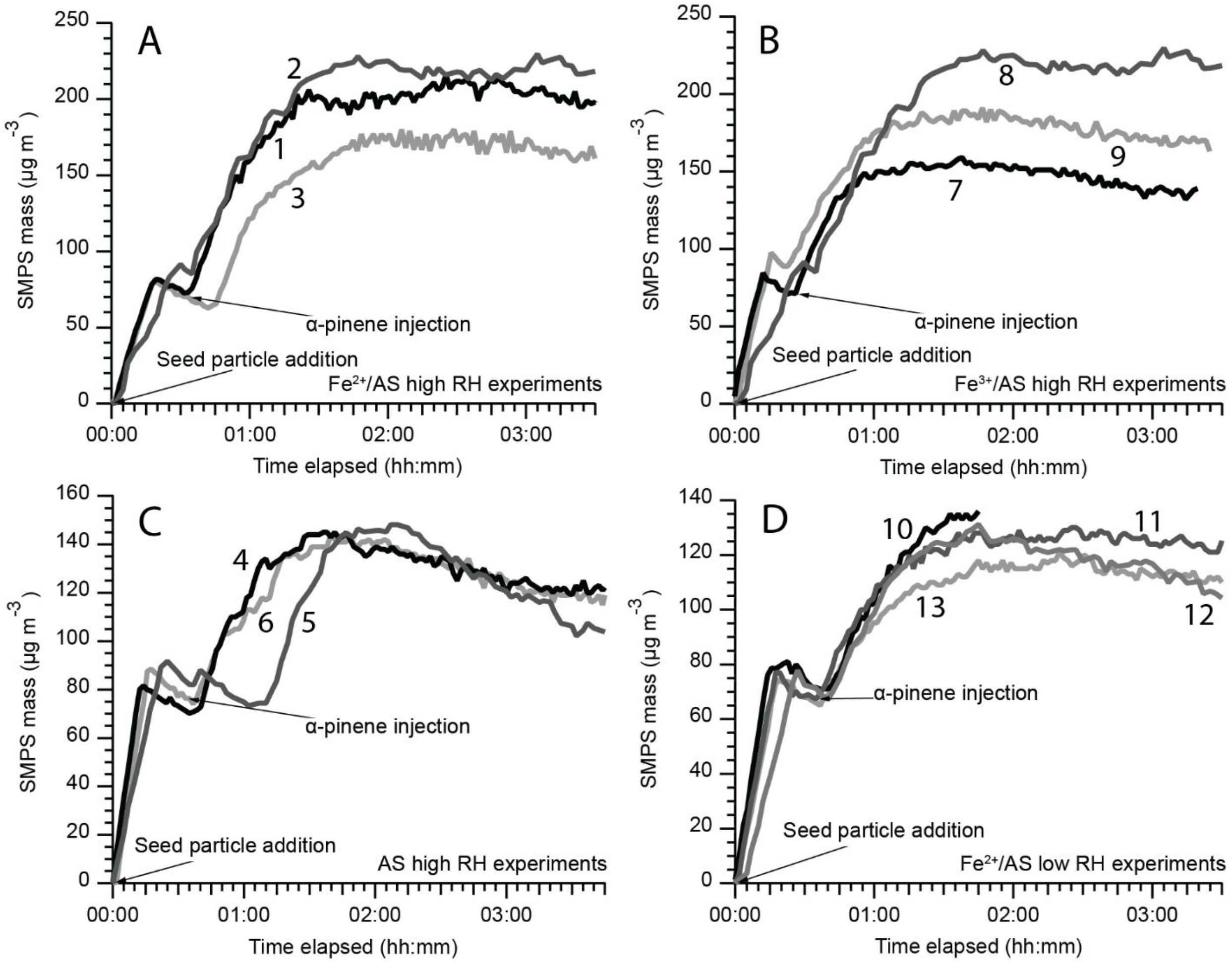

Figure S2. Time series of aerosol mass (in μg m^{-3}) measured by a scanning mobility particle sizer (SMPS) for all experiments conducted at high RH with (A) Fe^{2+}/AS, (B) Fe^{3+}/AS, and (C) AS seed particles, and at low RH with (D) Fe^{2+}/AS seed particles. The replicate experiments are denoted as different gray scale traces. Time 00:00 h shows when seed particles were injected to the chamber as seen by the initial increase in aerosol mass. This was followed at ~00:30 h by the injection of α-pinene into the chamber, which commenced the start of SOA growth and resulted in the increase in aerosol mass between ~00:30 and 01:30 h. Aerosol mass was calculated assuming a constant density of 1.2 g cm^{-3} (α-pinene). The numbers next to the traces represent the associated experiment number listed in Table S1. The uncertainties for the SMPS measurements are ±20%.

S4. PEARSON CORRELATION COEFFICIENT

The linear correlation between experiments was evaluated by calculating the Pearson correlation coefficient for selected monomers and dimers between every experiment using R Statistical Software (version 4.3.1).[1] To evaluate the correlation of replicate measurements done under the same experimental conditions, a comparison of extractive electrospray ionization time-of-flight mass spectrometer (EESI-ToF) data was performed for data collected 1 h after the addition of α-pinene, when aerosol mass had reached a maximum, i.e., T2 (Figure 1). The results were then visualized using the corrplot R package (v0.92),[2] and are shown in Figure S1. Replicate experiments are shown as individual 'squares', where the color denotes the Pearson correlation coefficient between two experiments during the same period of secondary organic aerosol (SOA) formation (T2). For example, a Pearson correlation coefficient of 1 implies that all data points fall exactly on a line, i.e., that the linear equation describing the relationship between X and Y fits perfectly. The darker the color the higher the degree of correlation between two experiments during the same period.

All experiments, regardless of seed type or RH demonstrated good correlation (>0.50), which is expected since all experiments utilized similar precursor concentrations (~50 ppb α-pinene and ~300 ppb O_3) resulting in the same initial gas-phase α-pinene oxidation products, which then partitioned into the condensed-phase to form SOA. As expected, replicate experiments of the same 'type' showed very strong correlation, which varied from ~0.8 for experiments conducted with AS seed particles at high RH to >0.95 for experiments with Fe^{2+}/AS seed at low RH. As such, the data from the same experiment 'type' were averaged and presented as such in the main manuscript.

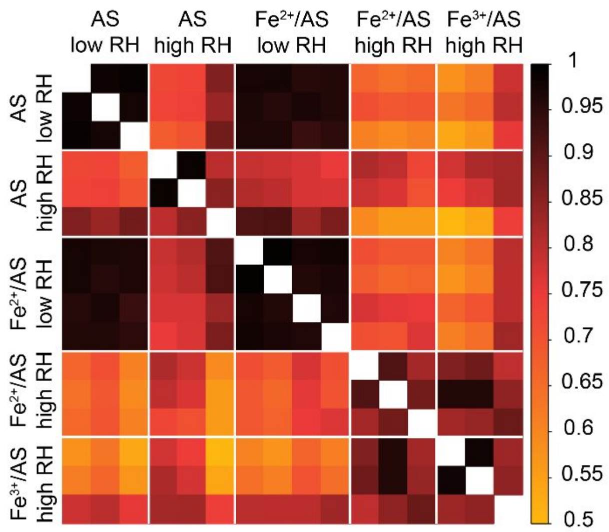

Figure S3. Pearson correlation plot of all experiments conducted with and without Fe-containing seed (denoted Fe^{2+}/AS and Fe^{3+}/AS or AS, respectively) at high and low RH. The color scale shows the associated Pearson correlation value.

S5. MONOMER AND DIMER TIMESERIES

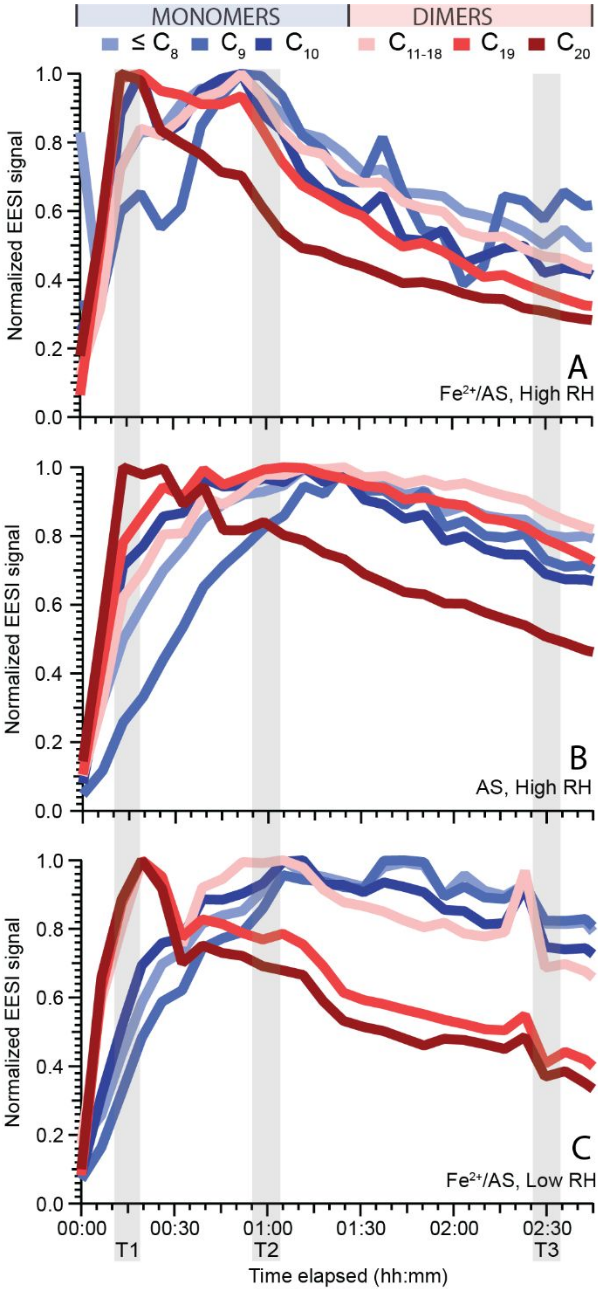

Figure S4. Time series of monomers and dimers during initial SOA formation, and subsequent aging for experiments conducted on: (A) Fe^{2+}/AS seed particles at high RH, (B) AS seed particles at high RH and (C) Fe^{2+}/AS seed particles at low RH. Monomers (blue shaded traces) and dimers (red shaded traces) are shown as a normalized EESI signal.

S6. DECAY RATES

An exponential fitting factor was used to determine the lifetime (τ, s) and average rate of decay (s^{-1}) for replicate experiments and is shown in Tables S2 and S3 for C_{19} and C_{20} dimers respectively.

Table S2. Average decay rate (s^{-1}) and lifetime (s) and their 1σ standard deviations, of selected C_{19} species for experiments conducted on seed particles containing Fe^{2+}/AS or AS at high and low RH. n/a = not applicable

	Fe^{2+}/AS high RH		AS high RH		Fe^{2+}/AS low RH	
Species	Decay rate ($\times 10^{-4}$ s^{-1})	τ ($\times 10^{4}$ s)	Decay rate ($\times 10^{-4}$ s^{-1})	τ ($\times 10^{4}$ s)	Decay rate ($\times 10^{-4}$ s^{-1})	τ ($\times 10^{4}$ s)
$C_{19}H_{30}O_4$	10±9	0.2±0.2	3±4	5±7	3±1	0.3±0.1
$C_{19}H_{30}O_5$	8±5	0.2±0.1	2±2	1±1	3±2	1±2
$C_{19}H_{30}O_6$	8±3	0.13±0.04	2±2	83±143	2±2	5±9
$C_{19}H_{32}O_5$	9±2	0.11±0.02	4±2	4±3	5±5	0.3±2
$C_{19}H_{32}O_6$	3±4	3±5	1.1±0.9	0.4±0.3	1±2	39±50
$C_{19}H_{32}O_7$	6±3	0.19±0.07	2.1±0.7	116±200	1±2	47±54
$C_{19}H_{32}O_8$	4±1	0.3±0.1	4±3	0.4±0.3	1±1	42±70
FeI^{+}	2±1	0.01±0.06	n/a	n/a	8±5	0.2±0. 1
SO_4^{2-}	1±1	4±6	3±4	50±50	0.0075± 0.0008	133±230

Table S3. Average decay rate (s^{-1}) and lifetime (s) and their 1σ standard deviations, of selected C_{20} species for experiments conducted on seed particles containing AS or Fe^{2+}/AS at high and low RH. n/a = not applicable

Species	Fe^{2+}/AS high RH		AS high RH		Fe^{2+}/AS low RH	
	Decay rate ($\times10^{-4}$ s^{-1})	τ ($\times10^{4}$ s)	Decay rate ($\times10^{-4}$ s^{-1})	τ ($\times10^{4}$ s)	Decay rate ($\times10^{-4}$ s^{-1})	τ ($\times10^{4}$ s)
$C_{20}H_{32}O_5$	10±6	0.2±.2	0.5±0.8	117±171	3.6±0.3	0.28±0.02
$C_{20}H_{32}O_6$	7±3	0.16±0.08	3±1	0.5±0.5	3±1	0.4±0.2
$C_{20}H_{32}O_7$	7±4	0.2±0.1	1.8±0.6	0.6±0.3	3±2	0.6±0.5
$C_{20}H_{32}O_8$	5±4	0.4±0.5	2±2	191±331	1±1	14±23
$C_{20}H_{32}O_9$	4±3	0.4±0.4	3±2	109±188	1±1	16±25
$C_{20}H_{32}O_{10}$	6.15± 0.09	0.163± 0.002	2±2	103±178	0.7±0.8	39±65
$C_{20}H_{32}O_{11}$	6	0.16	2±2	100±173	1±1	18±18
$C_{20}H_{34}O_4$	10	0.09	2±1	0.9±0.8	4±4	19±32
$C_{20}H_{34}O_5$	n/a	n/a	6.0±0.6	55±93	3±1	0.3±0.1
$C_{20}H_{34}O_6$	8±5	0.2±0.1	2±1	0.6±0.5	3±1	0.4±0.2
$C_{20}H_{34}O_7$	7±1	0.14±0.02	3±1	0.4±0.1	2±1	3±4
$C_{20}H_{34}O_8$	9±5	0.14±0.08	4±2	0.4±0.2	2.4±0.7	0.4±0.2
$C_{20}H_{34}O_9$	9±3	0.11±0.04	2±1	0.5±0.2	2±1	2±3
$C_{20}H_{34}O_{10}$	9±1	0.10±0.01	3±1	0.35±0.04	2.7±0.4	4±6
FeI^{+}	2±1	0.01±0.06	n/a	n/a	8±5	0.2±0.1
SO_4^{2-}	1±1	4±6	3±4	50±50	0.0075± 0.0008	133±230

Figure S5 depicts the decay rate ratio for experiments conducted on Fe^{2+}/AS seed at low RH and experiments conducted on AS seed particles at high RH. With the exception of $C_{20}H_{32}O_5$ and $C_{20}H_{34}O_5$, the ratio of other C_{19} and C_{20} dimers fell at or below the 1:1 AS ratio, indicating their rate of decay was slower for experiments conducted on Fe^{2+}/AS seed at low RH, than for AS seed experiments at high RH. This is contrary to what is shown in Figure 4 (main manuscript), and suggests that the reaction of Fe with peroxide containing dimers may be limited by microphysical aerosol properties such phase[10] and mixing[11] state.

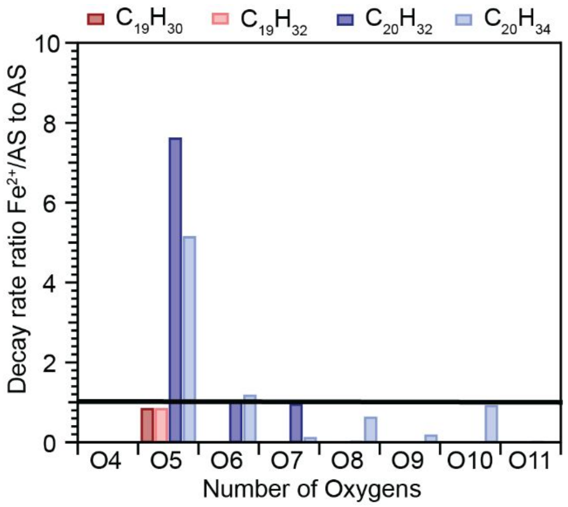

Figure S5. Ratio between decay fits for C_{19} (red bars) and C_{20} (blue bars) dimers vs. their number of oxygen (x-axis) for low RH Fe^{2+}/AS and high RH AS high experiments. The black bar represents the self-normalized 1:1 ratio for AS experiments, i.e., points falling above this line represent dimers that decayed faster in the presence of Fe at low RH, than in its absence at high RH.

S7. AGING OF FE CONTAINING SOA

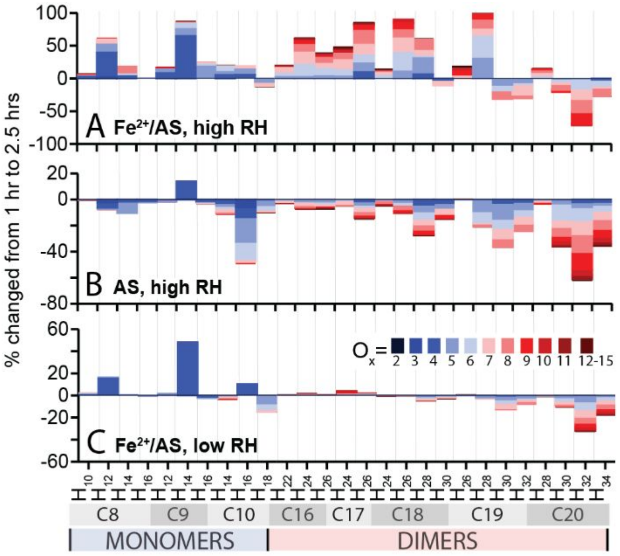

Figure S6: Bar graphs showing relative percent changes in key monomers and dimers between ~1 h (T2) and 2.5 h (T3) post α-pinene injection for different experimental scenarios of (A) Fe^{2+}/AS seed particles at high RH, (B) AS seed particles at high RH and (C) Fe^{2+}/AS seed particles at low high. Increases in relative signal are denoted as positive values, whereas decreases are denoted as negative values. EESI-ToF data were normalized to EESI-ToF SO_4^{2-} signal and SMPS aerosol mass to account for changes in signal due to wall losses over time.

S8. T3 BAR GRAPH LOW RH FE^{2+}/AS CASE

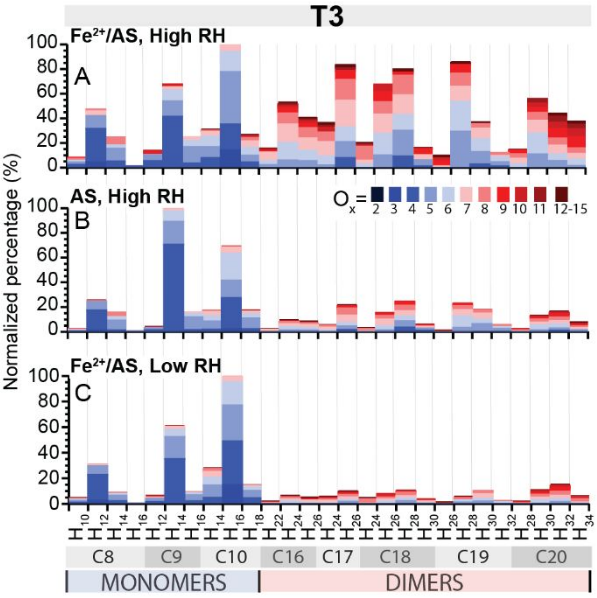

Figure S7: Bar graphs of key monomers and dimers for experiments conducted at high RH on (A) Fe^{2+}/AS, (B) AS seed particles, and (C) low RH on Fe^{2+}/AS seed particles, at ~2.5 h following the addition of α-pinene to the chamber (T3, Figure 1).

S9. FE^{3+}/AS EXPERIMENTS

Experiments conducted at high RH using Fe^{3+}/AS seed particles are summarized in Table S1. An overview of key data for these experiments is shown below. Overall, SOA generated at high RH on Fe^{3+}/AS seed particles exhibited strong correlation ($R > 0.8$, Figure S1) with those generated on Fe^{2+}/AS seed particles, and similar to the correlation between replicate measurements of the same seed types.

Figure S8 shows time series of the average SOA mass concentration generated via dark ozonolysis of α-pinene in the presence of Fe^{3+}/AS seed particle types, at high humidity. Maximum SOA mass concentration was observed ~1 h after α-pinene addition, as with the other experiments shown in Figure 1. In the presence of Fe^{3+}/AS seeds at high RH, a maximum SOA mass of ~88 µg m^{-3} formed, which was higher than the ~60 µg m^{-3} formed in the absence of Fe/at low RH, but lower than the ~120 µg m^{-3} formed in the presence of Fe^{2+}/AS.

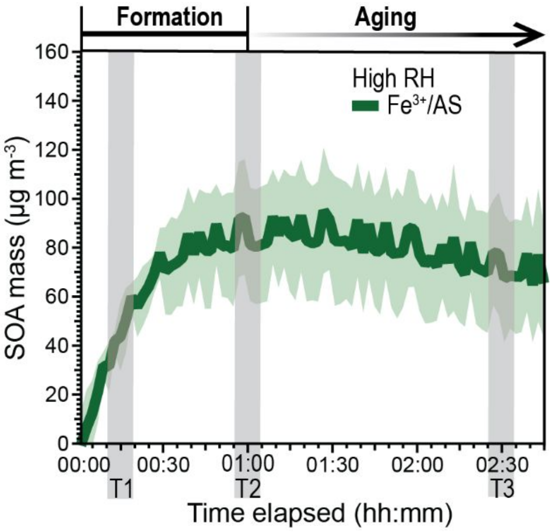

Figure S8: Time series of average aerosol mass (in µg m^{-3}) generated from the dark ozonolysis of α-pinene in the presence of Fe^{3+}/AS seed particles at high RH. Variation in the replicate experiments is shown as ± 1 standard deviation (shaded region). Gray shaded regions labeled

T1, T2 and T3 represent case study periods. A summary of SOA mass formed for all experiments is in Table S1.

Key monomers and dimers present in SOA at T1 are shown in Figure S9. Similar to the other experimental cases shown in Figure 2 of the main manuscript, SOA generated on Fe^{3+}/AS were dominated by $C_{9,10}$ monomers and $C_{19,20}$ dimers. Furthermore both Fe^{3+}/AS and Fe^{2+}/AS experiments exhibited many similarities. However, for the Fe^{3+}/AS experiments a higher fraction of peroxide containing $C_{10}H_{18}$ monomers was observed, potentially due to the reduced presence or absence of Fe^{2+} which is expected to react with peroxides, i.e., through R4 (main manuscript).

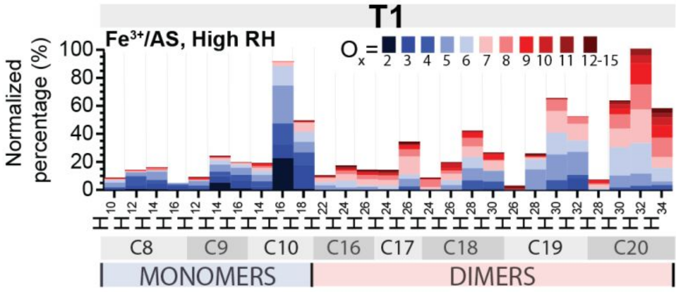

Figure S9. Bar graph of key monomers and dimers for experiments conducted at high RH on Fe^{3+}/AS seed particles, at ~10 min following the addition of α-pinene to the chamber (T1, Figure 1). Bar graphs for T2 and T3 are shown in Figure 5 in the main manuscript.

Similar again to the Fe^{2+}/AS case, SOA formed on Fe^{3+}/AS seed particles exhibited a higher fraction of dimers to monomers, where each represented ~70% and ~30% of total monomer and dimers respectively (Figure S10). However, unlike the Fe^{2+}/AS seed experiments where the monomer/dimer ratio remained stable over the course of the experiments, Fe^{3+}/AS experiments exhibited a very slight decrease in dimers (68% at T1 to 62% at T3); potentially due to reduced Fe^{2+} availability driven by slower or reduced reduction of Fe^{3+} to Fe^{2+}.

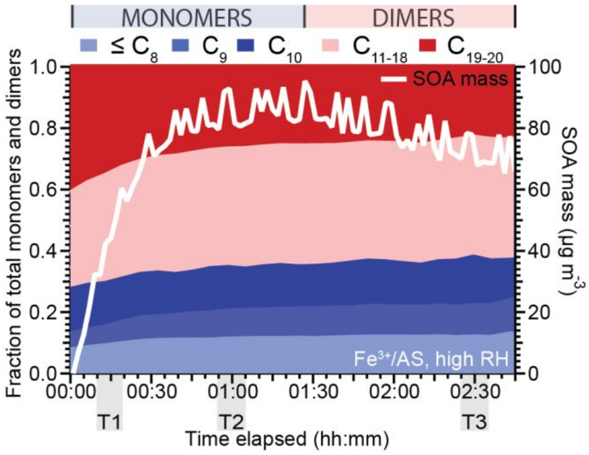

Figure S10. Time series for monomers (blue) and dimers (red) for experiments conducted at high RH on Fe^{3+}/AS seed particles. Monomers and dimers are shown as a fraction of the total mass flux. The average SOA mass concentration is also shown as the white trace.

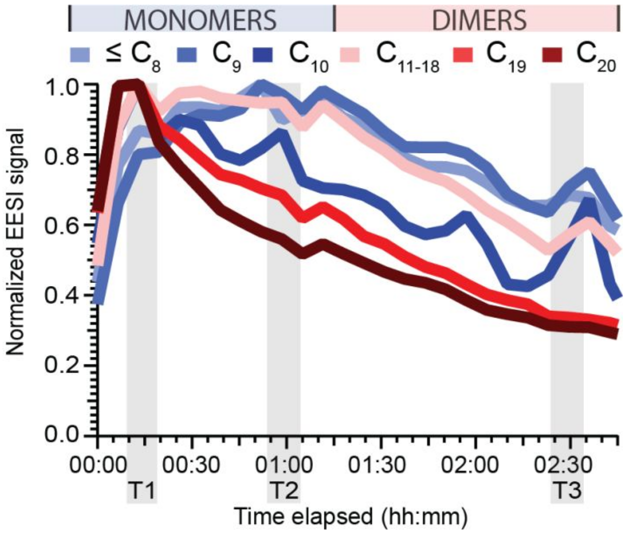

Figure S11. Time series of monomers and dimers during initial SOA formation, and subsequent aging for experiments conducted on Fe^{3+}/AS seed particles at high RH. Monomers (blue shaded traces) and dimers (red shaded traces) are shown as a normalized EESI signal.

S10. COMPOSITION OF FE^{2+}/AS EXPERIMENTS AT DIFFERENT RHS

To explore the potential impact of this chemistry under a broader range of relative humidities, an experiment was conducted at ~50% RH with Fe^{2+}/AS seed particles. As with the high RH experiments, SOA formed under intermediate RH conditions were found to contain a large fraction of dimers compared to monomers.

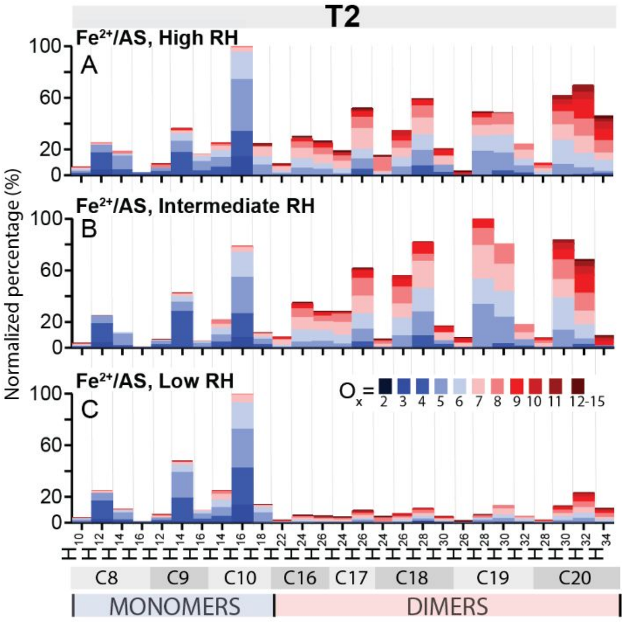

Figure S12. Bar graph of key monomers and dimers for experiments conducted at (A) high RH (B) intermediate RH and (C) low RH on Fe^{2+}/AS seed particles, at ~1 hr following the addition of α-pinene to the chamber (T2, Figure 1).

REFERENCES

(1) *R: A Language and Environment for Statistical Computing*; 2023. https://www.R-project.org/

(2) *R package 'corrplot': Visualization of a Correlation Matrix*; 2021. https://github.com/taiyun/corrplot

(3) Wolfe, G. M.; Marvin, M. R.; Roberts, S. J.; Travis, K. R.; Liao, J. The Framework for 0-D Atmospheric Modeling (F0AM) v3.1. *Geosci. Model Dev.* **2016**, *9* (9), 3309-3319; DOI: 10.5194/gmd-9-3309-2016.

(4) Jenkin, M. E.; Saunders, S. M.; Pilling, M. J. The tropospheric degradation of volatile organic compounds: a protocol for mechanism development. *Atmos. Environ.* **1997**, *31* (1), 81-104; DOI: 10.1016/S1352-2310(96)00105-7.

(5) Saunders, S. M.; Jenkin, M. E.; Derwent, R. G.; Pilling, M. J. Protocol for the development of the Master Chemical Mechanism, MCM v3 (Part A): tropospheric degradation of non-aromatic volatile organic compounds. *Atmos. Chem. Phys.* **2003**, *3* (1), 161-180; DOI: 10.5194/acp-3-161-2003.

(6) Saunders, S. M.; Jenkin, M. E.; Derwent, R. G.; Pilling, M. J. Protocol for the development of the Master Chemical Mechanism, MCM v3 (Part A): tropospheric degradation of non-aromatic volatile organic compounds. *Atmos. Chem. Phys.* **2003**, *3*, 161-180; DOI: 10.5194/acp-3-161-2003.

(7) Jenkin, M. E.; Saunders, S. M.; Wagner, V.; Pilling, M. J. Protocol for the development of the Master Chemical Mechanism, MCM v3 (Part B): tropospheric degradation of aromatic volatile organic compounds. *Atmos. Chem. Phys.* **2003**, *3*, 181-193; DOI: 10.5194/acp-3-161-2003.

(8) Bloss, C.; Wagner, V.; Jenkin, M. E.; Volkamer, R.; Bloss, W. J.; Lee, J. D.; Heard, D. E.; Wirtz, K.; Martin-Reviejo, M.; Rea, G.; Wenger, J. C.; Pilling, M. J. Development of a detailed

chemical mechanism (MCMv3.1) for the atmospheric oxidation of aromatic hydrocarbons. *Atmos. Chem. Phys.* **2005**, *5* (3), 641-664; DOI: 10.5194/acp-5-641-2005.

(9) Jenkin, M. E.; Wyche, K. P.; Evans, C. J.; Carr, T.; Monks, P. S.; Alfarra, M. R.; Barley, M. H.; McFiggans, G. B.; Young, J. C.; Rickard, A. R. Development and chamber evaluation of the MCM v3.2 degradation scheme for β-caryophyllene. *Atmos. Chem. Phys.* **2012**, *12* (11), 5275-5308; DOI: 10.5194/acp-12-5275-2012.

(10) Reid, J. P.; Bertram, A. K.; Topping, D. O.; Laskin, A.; Martin, S. T.; Petters, M. D.; Pope, F. D.; Rovelli, G. The viscosity of atmospherically relevant organic particles. *Nat. Comm.* **2018**, *9* (1), 956; DOI: 10.1038/s41467-018-03027-z.

(11) Riemer, N.; Ault, A. P.; West, M.; Craig, R. L.; Curtis, J. H. Aerosol Mixing State: Measurements, Modeling, and Impacts. *Rev. Geophys.* **2019**, *57* (2), 187-249; DOI: 10.1029/2018rg000615.
